# *Lactobacillus reuteri* AN417 cell-free culture supernatant as a novel antibacterial agent targeting oral pathogenic bacteria

**DOI:** 10.1038/s41598-020-80921-x

**Published:** 2021-01-15

**Authors:** Kyung Mi Yang, Ji-Sun Kim, Hye-Sung Kim, Young-Youn Kim, Jeong-Kyu Oh, Hye-Won Jung, Doo-Sang Park, Kwang-Hak Bae

**Affiliations:** 1 Institute of Biomedical Science, Apple Tree Dental Hospital, 1450, Jungang-ro, Ilsanseo-gu, Goyang-si, Gyeonggi-do 10387 Republic of Korea; 2 Biological Resources Center, Korea Research Institute of Bioscience & Biotechnology (KRIBB), Jeong-up, 56212 Republic of Korea

**Keywords:** Microbiology, Antimicrobials

## Abstract

*Lactobacillus reuteri* AN417 is a newly characterized probiotic strain. The activity of AN417 against oral pathogenic bacteria is unknown. We investigated the antibacterial activity of cell-free *L. reuteri* AN417 culture supernatant (LRS) against three oral pathogens: *Porphyromonas gingivalis*, *Fusobacterium nucleatum*, and *Streptococcus mutans*. *P. gingivalis* and *F. nucleatum* have been implicated in periodontal disease, whereas *S. mutans* causes dental caries. Exposing these oral pathogenic bacteria to LRS significantly reduced their growth rates, intracellular ATP levels, cell viability, and time-to-kill. The minimal inhibitory volume of LRS was 10% (v/v) against *P. gingivalis*, 20% (v/v) for *F. nucleatum*, and 30% (v/v) for *S. mutans*. LRS significantly reduced the integrity of biofilms and significantly suppressed the expression of various genes involved in *P. gingivalis* biofilm formation. The *L. reuteri* AN417 genome lacked genes encoding reuterin, reuteran*,* and reutericyclin, which are major antibacterial compounds produced in *L. reuteri* strains. LRS treated with lipase and α-amylase displayed decreased antibacterial activity against oral pathogens. These data suggest that the antibacterial substances in LRS are carbohydrates and/or fatty acid metabolites. Our results demonstrate that LRS has antimicrobial activity against dental pathogenic bacteria, highlighting its potential utility for the prevention and treatment of *P. gingivalis* periodontal disease.

## Introduction

Periodontal disease is a chronic inflammatory disease caused by the accumulation of different pathogenic biofilm-forming bacteria in dental pockets^1^. Biofilms that develop on tooth surfaces contain oral microbes, and the formation of bacterial plaque causes gingivitis with redness and swelling of the gingiva^2^. Periodontitis refers to an irreversible loss of adhesion, in which tissues such as periodontal ligaments, alveolar bone, and chalk that support teeth are absorbed as the inflammatory response of the tissues to pathogenic bacterial stimulation intensifies^3^. Collagen fibers in the periodontal ligament are destroyed, forming a periodontal sac between the gingiva and the teeth, deepening through the absorption of the alveolar bone^3,4^. This creates an ecological environment that favors the propagation of various anaerobic bacteria, causing periodontal disease^5^. Endotoxins or metabolites formed by microorganisms participate in tissue breakthrough processes by increasing the secretion of pro-inflammatory cytokines from tissues and immune cells^6^.

The pathogens most known to be associated with the development of oral diseases are *Porphyromonas gingivalis*, *Fusobacterium nucleatum*, and *Streptococcus mutans*. Among oral pathogens, *P. gingivalis* and *F. nucleatum* are Gram-negative and obligate anaerobes that cause periodontal diseases^7,8^. These two oral pathogens degrade collagen, induce halitosis, and produce endotoxins, such as lipopolysaccharides (LPS), which destroy alveolar bone and cause tooth loss^9^. *S. mutans* produces acidic compounds and plays an important role in the formation of biofilms on teeth, which cause dental caries^10^. These oral pathogens can penetrate directly into the vascular endothelial cells or enter damaged blood vessels and adhere to specific organs, ultimately leading to systemic disease^11^.

Substances produced by lactic acid bacteria during the metabolism of prebiotics (dietary fiber) can be beneficial to human health^12^. In a rat model, the administration of live probiotic *Bifidobacteria* was reported to protect against periodontal destruction and to decrease inflammatory intermediates in rats with ligature-induced periodontitis^13^. A recent review highlighted potential antimicrobial agents, including those produced by lactic acid bacteria, for the treatment of dental diseases^4^.

Although antibiotics are still widely used to treat oral diseases, they cause side effects, such as the generation of resistant strains and microbial substitution^14^. Thus, a variety of natural substances that have few side effects are being used proactively. Numerous ongoing studies are examining the antimicrobial activities of plant extracts that inhibit the growth of oral pathogenic bacteria^15–17^. Adjuvant treatments using ozone are also being explored as a new approach for managing chronic periodontitis^18^.

Recent data have indicated the potential value of probiotic microorganisms in oral health^19^. For example, cell-free culture supernatants of a *Weissella cibaria* strain showed antibacterial activity against periodontal pathogens. The effects were dependent on either the acidity of the supernatant or the level of hydrogen peroxide produced by *W. cibaria*^20^. Another study reported antimicrobial and antibiofilm activities of *Lactobacillus kefiranofaciens* culture supernatants against oral pathogens. Heat-killed *L. reuteri* and supernatants from cell-free *L. reuteri* cultures had antibacterial effects on *P. gingivalis* that were similar to those of live *L. reuteri* cells, although in an invertebrate model, hemocyte density was significantly increased only in the presence of live *L. reuteri* and not with the culture supernatant or heat-killed cells^21^. During fermentation, lactic acid bacteria produce metabolites, such as extracellular polymeric substances, functional proteins, and peptides, which are bioactive compounds with known beneficial effects on human health^22^. The antimicrobial effect of the supernatant of lactic acid bacteria (LAB) was not after treatment with proteinase K, pepsin, and papain^23^.

This study evaluated the antibacterial potential of a new probiotic strain, *L. reuteri* AN417, to improve oral health. Our findings indicate that this strain has potential as a therapeutic agent for the treatment of chronic periodontitis and the prevention of dental caries.

## Results

### Isolation and characterization of *L. reuteri* strains

**Figure 1:**
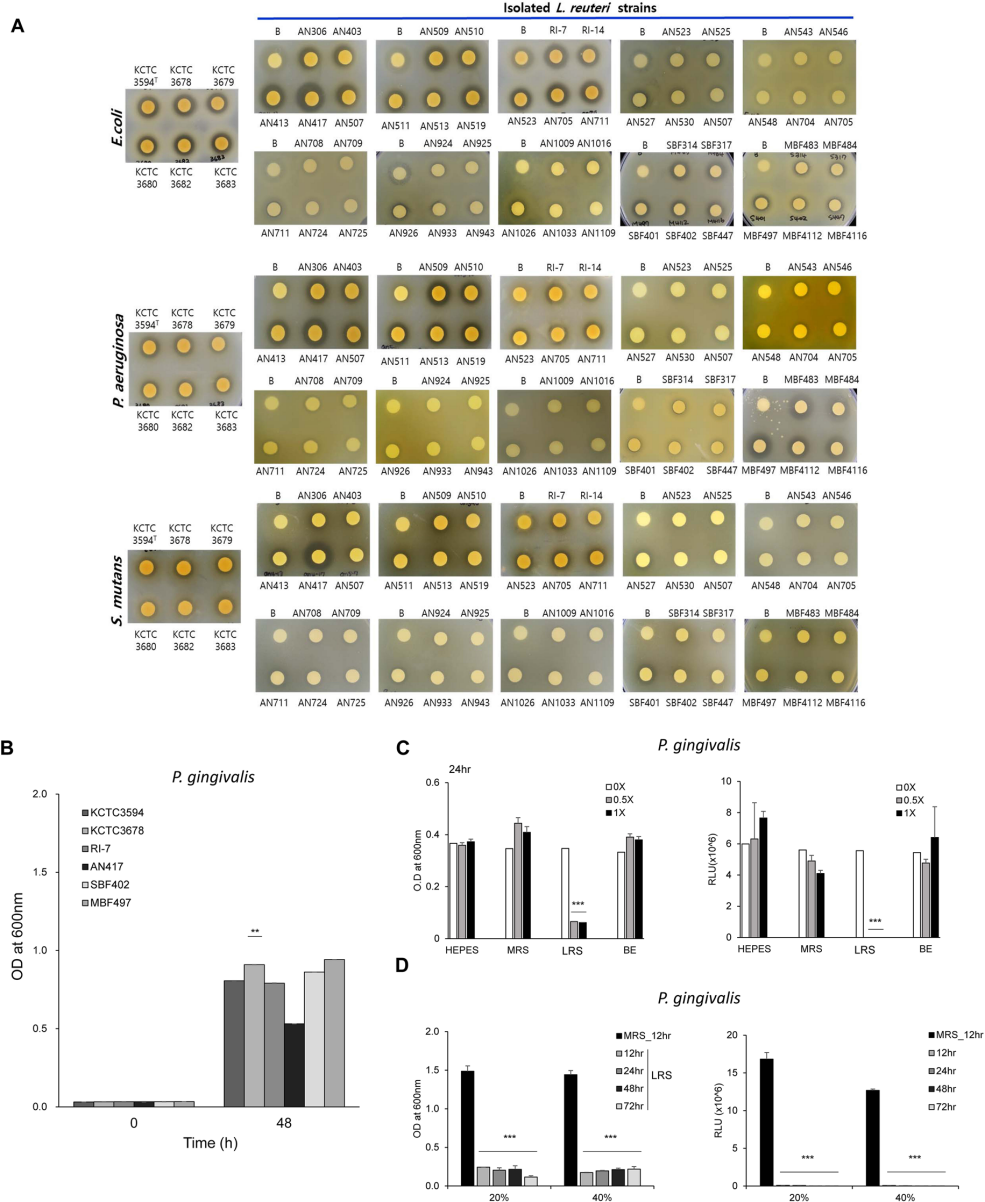
Evaluation of the antimicrobial activity of culture supernatants of *L. reuteri* strains against *E. coli, P. aeruginosa, S. mutans*, and *P. gingivalis.* (**A**) The antimicrobial activity of culture supernatants from newly isolated and reference *L. reuteri* strains against *E. coli, P. aeruginosa* and *S. mutans* was analyzed through disk diffusion assays following 24 h incubation at 37 °C. (**B**) Growth curve of *P. gingivalis* at OD_600_ measured using a disposable curvet 48 h after treatment with the supernatants of each strains. Significant differences from the control (*p* < 0.01) are indicated by **. (**C**) The effect of various concentrations of LRS and BE on *P. gingivalis* growth, measured by OD_600_ (left panel) and intracellular ATP levels (right panel) following a 24 h incubation at 37 °C. MRS medium was used as the negative control. Significant differences from the control (*p* < 0.001) are indicated by ***. (**D**) The effect of 20% (v/v) and 40% (v/v) LRS on *P. gingivalis* growth (left panel) and intracellular ATP levels (right panel) over time. MRS medium was used as the negative control. Significant differences from the control (*p* < 0.001) are indicated by ***.

We isolated 135 *L. reuteri* strains from human infants and 6-month-old female swine under anaerobic conditions. Bacterial isolates were identified by 16S rRNA gene sequencing and a matrix-assisted laser desorption/ionization time of flight (MALDI-TOF) biotyper (Bruker, Table 1). Primary screening for antimicrobial activity against pathogens, including *Escherichia coli* (KCTC 2571), *Pseudomonas aeruginosa* (DSM 50071), and *S. mutans* (KCTC 3065), was performed to select *L. reuteri* strains that had exhibited antimicrobial activity against periodontopathic bacteria in a disk diffusion assay (Fig. 1A). The results showed that *L. reuteri* AN417 displayed the strongest antibacterial activity against pathogens.

**Table 1:** Isolation of *Lactobacillus reuteri* strains, and determination of 1,3-PDO and reuterin production. 1,3-PDO and reuterin production were determined in *Lactobacillus reuteri* isolates using HPLC and Colorimetric method, respectively.

No	ID	Strain	Origin	Residual glucose (g/L)	Residual glycerol (g/L)	1,3-PDO production (g/L)^a^	Reuterin production^b^
1	PB3	*Lactobacillus reuteri*	Swine feces	1.96	6.93	++	+
2	PB6	*Lactobacillus reuteri*	Swine feces	0.16	5.51	+++	+
3	PF4	*Lactobacillus reuteri*	Swine feces	1.18	4.96	+++	–
4	PPF3	*Lactobacillus reuteri*	Swine feces	2.76	8.10	++	–
5	PMA2	*Lactobacillus reuteri*	Swine feces	0.05	21.49	–	–
6	PMF1	*Lactobacillus reuteri*	Swine feces	0.64	20.99	–	–
7	PMF2	*Lactobacillus reuteri*	Swine feces	0.57	21.45	–	–
8	AN306	*Lactobacillus reuteri*	Small intestine of swine	0.35	21.47	–	–
9	AN313	*Lactobacillus reuteri*	Small intestine of swine	0.56	20.84	–	–
10	AN403	*Lactobacillus reuteri*	Small intestine of swine	4.51	21.47	–	–
11	AN413	*Lactobacillus reuteri*	Small intestine of swine	0.05	6.05	–	–
12	AN417	*Lactobacillus reuteri*	Small intestine of swine	1.08	20.40	–	–
13	AN507	*Lactobacillus reuteri*	Small intestine of swine	0.45	20.09	–	–
14	AN509	*Lactobacillus reuteri*	Small intestine of swine	0.08	21.43	–	–
15	AN510	*Lactobacillus reuteri*	Small intestine of swine	2.21	21.50	–	–
16	AN511	*Lactobacillus reuteri*	Small intestine of swine	0.28	21.42	–	–
17	AN513	*Lactobacillus reuteri*	Small intestine of swine	5.19	21.26	–	–
18	AN516	*Lactobacillus reuteri*	Small intestine of swine	1.39	21.48	–	–
19	AN519	*Lactobacillus reuteri*	Small intestine of swine	0.20	21.30	–	–
20	AN521	*Lactobacillus reuteri*	Small intestine of swine	0.18	21.17	–	–
21	AN523	*Lactobacillus reuteri*	Small intestine of swine	1.23	21.30	–	–
22	AN525	*Lactobacillus reuteri*	Small intestine of swine	0.13	21.28	–	–
23	AN527	*Lactobacillus reuteri*	Small intestine of swine	0.12	20.95	–	–
24	AN530	*Lactobacillus reuteri*	Small intestine of swine	0.46	21.21	–	–
25	AN540	*Lactobacillus reuteri*	Small intestine of swine	1.00	21.36	–	–
26	AN543	*Lactobacillus reuteri*	Small intestine of swine	0.10	21.42	–	–
27	AN546	*Lactobacillus reuteri*	Small intestine of swine	3.58	21.42	–	–
28	AN548	*Lactobacillus reuteri*	Small intestine of swine	0.24	21.32	–	–
29	AN703	*Lactobacillus reuteri*	Small intestine of swine	8.13	21.46	–	–
30	AN704	*Lactobacillus reuteri*	Small intestine of swine	0.42	21.37	–	–
31	AN705	*Lactobacillus reuteri*	Small intestine of swine	1.38	20.89	–	–
32	AN708	*Lactobacillus reuteri*	Small intestine of swine	5.99	21.38	–	–
33	AN709	*Lactobacillus reuteri*	Small intestine of swine	3.36	21.42	–	–
34	AN711	*Lactobacillus reuteri*	Small intestine of swine	2.64	21.41	–	–
35	AN722	*Lactobacillus reuteri*	Small intestine of swine	4.81	21.24	–	–
36	AN724	*Lactobacillus reuteri*	Small intestine of swine	0.12	21.34	–	–
37	AN727	*Lactobacillus reuteri*	Small intestine of swine	2.48	21.22	–	–
38	AN832	*Lactobacillus reuteri*	Small intestine of swine	7.69	21.39	–	–
39	AN903	*Lactobacillus reuteri*	Small intestine of swine	1.03	21.47	–	–
40	AN904	*Lactobacillus reuteri*	Small intestine of swine	3.63	21.49	–	–
41	AN906	*Lactobacillus reuteri*	Small intestine of swine	9.73	18.93	–	–
42	AN919	*Lactobacillus reuteri*	Small intestine of swine	12.51	18.90	–	–
43	AN924	*Lactobacillus reuteri*	Small intestine of swine	7.59	18.78	–	–
44	AN925	*Lactobacillus reuteri*	Small intestine of swine	9.31	19.01	–	–
45	AN926	*Lactobacillus reuteri*	Small intestine of swine	9.90	21.05	–	–
46	AN933	*Lactobacillus reuteri*	Small intestine of swine	4.88	19.54	–	–
47	AN941	*Lactobacillus reuteri*	Small intestine of swine	10.13	19.27	–	–
48	AN943	*Lactobacillus reuteri*	Small intestine of swine	0.75	20.79	–	–
49	AN947	*Lactobacillus reuteri*	Small intestine of swine	9.92	18.72	–	+
50	AN1002	*Lactobacillus reuteri*	Small intestine of swine	12.09	19.00	–	–
51	AN1009	*Lactobacillus reuteri*	Small intestine of swine	9.69	18.99	–	–
52	AN1016	*Lactobacillus reuteri*	Small intestine of swine	4.47	20.87	–	–
53	AN1019	*Lactobacillus reuteri*	Small intestine of swine	8.83	20.52	–	–
54	AN1026	*Lactobacillus reuteri*	Small intestine of swine	7.55	19.04	–	–
55	AN1033	*Lactobacillus reuteri*	Small intestine of swine	2.38	20.96	–	–
56	AN1034	*Lactobacillus reuteri*	Small intestine of swine	5.58	19.42	–	–
57	AN1109	*Lactobacillus reuteri*	Small intestine of swine	8.60	21.03	–	–
58	SBF301	*Lactobacillus reuteri*	Infant feces	0.51	5.70	++	+
59	SBF302	*Lactobacillus reuteri*	Infant feces	1.30	7.37	++	+
60	SBF303	*Lactobacillus reuteri*	Infant feces	1.04	7.09	++	++
61	SBF304	*Lactobacillus reuteri*	Infant feces	2.31	8.33	++	+
62	SBF305	*Lactobacillus reuteri*	Infant feces	10.85	15.80	+	+
63	SBF306	*Lactobacillus reuteri*	Infant feces	0.32	5.98	+++	+
64	SBF307	*Lactobacillus reuteri*	Infant feces	0.59	6.27	+++	++
65	SBF309	*Lactobacillus reuteri*	Infant feces	3.99	9.26	++	–
66	SBF310	*Lactobacillus reuteri*	Infant feces	3.57	9.09	++	+
67	SBF311	*Lactobacillus reuteri*	Infant feces	0.08	5.20	++	++
68	SBF312	*Lactobacillus reuteri*	Infant feces	4.47	10.15	++	+
69	SBF313	*Lactobacillus reuteri*	Infant feces	7.43	12.87	++	+
70	SBF314	*Lactobacillus reuteri*	Infant feces	5.78	11.30	++	+
71	SBF315	*Lactobacillus reuteri*	Infant feces	4.40	9.93	++	+
72	SBF316	*Lactobacillus reuteri*	Infant feces	1.33	8.92	++	–
73	SBF317	*Lactobacillus reuteri*	Infant feces	12.47	16.74	+	–
74	SBF318	*Lactobacillus reuteri*	Infant feces	5.78	11.44	++	+
75	SBF320	*Lactobacillus reuteri*	Infant feces	11.91	16.46	+	+
76	SBF321	*Lactobacillus reuteri*	Infant feces	9.37	14.92	+	+
77	SBF325	*Lactobacillus reuteri*	Infant feces	0.32	5.27	+++	+
78	SBF326	*Lactobacillus reuteri*	Infant feces	6.18	11.47	++	+
79	SBF327	*Lactobacillus reuteri*	Infant feces	5.54	10.86	++	+++
80	SBF328	*Lactobacillus reuteri*	Infant feces	0.95	14.94	+	+++
81	SBF329	*Lactobacillus reuteri*	Infant feces	1.13	7.73	+++	–
82	SBF330	*Lactobacillus reuteri*	Infant feces	8.63	14.70	+	+
83	SBF331	*Lactobacillus reuteri*	Infant feces	0.39	6.30	+++	++
84	SBF332	*Lactobacillus reuteri*	Infant feces	0.77	7.17	+++	++
85	SBF333	*Lactobacillus reuteri*	Infant feces	1.59	8.07	+++	+++
86	SBF334	*Lactobacillus reuteri*	Infant feces	1.65	8.12	++	++
87	SBF335	*Lactobacillus reuteri*	Infant feces	0.32	6.50	+++	+++
88	SBF336	*Lactobacillus reuteri*	Infant feces	4.58	10.57	++	++
89	SBF401	*Lactobacillus reuteri*	Infant feces	0.29	6.41	+++	+
90	SBF402	*Lactobacillus reuteri*	Infant feces	1.27	9.55	++	++
91	SBF417	*Lactobacillus reuteri*	Infant feces	0.53	7.07	+++	+
92	SBF418	*Lactobacillus reuteri*	Infant feces	5.69	11.51	++	+
93	SBF419	*Lactobacillus reuteri*	Infant feces	0.32	5.36	+++	+
94	SBF420	*Lactobacillus reuteri*	Infant feces	0.29	6.19	+++	+
95	SBF447	*Lactobacillus reuteri*	Infant feces	0.66	6.86	+++	+
96	MBF301	*Lactobacillus reuteri*	Infant feces	0.99	7.02	+++	+
97	MBF302	*Lactobacillus reuteri*	Infant feces	0.25	5.39	+++	+
98	MBF303	*Lactobacillus reuteri*	Infant feces	0.47	5.20	+++	+
99	MBF304	*Lactobacillus reuteri*	Infant feces	0.41	4.18	+++	+
100	MBF307	*Lactobacillus reuteri*	Infant feces	12.83	17.83	+	+
101	MBF312	*Lactobacillus reuteri*	Infant feces	1.38	7.84	+++	+
102	MBF326	*Lactobacillus reuteri*	Infant feces	0.29	5.54	+++	+
103	MBF327	*Lactobacillus reuteri*	Infant feces	7.63	13.25	++	+
104	MBF329	*Lactobacillus reuteri*	Infant feces	12.96	17.58	+	+
105	MBF335	*Lactobacillus reuteri*	Infant feces	0.26	4.86	+++	+
106	MBF342	*Lactobacillus reuteri*	Infant feces	0.25	4.87	+++	+
107	MBF344	*Lactobacillus reuteri*	Infant feces	0.27	5.55	+++	+
108	MBF351	*Lactobacillus reuteri*	Infant feces	0.27	5.49	+++	+
109	MBF355	*Lactobacillus reuteri*	Infant feces	0.25	4.77	+++	+
110	MBF356	*Lactobacillus reuteri*	Infant feces	0.28	4.76	+++	+
111	MBF358	*Lactobacillus reuteri*	Infant feces	0.49	5.88	+++	+
112	MBF359	*Lactobacillus reuteri*	Infant feces	0.62	6.25	+++	+
113	MBF483	*Lactobacillus reuteri*	Infant feces	2.82	9.16	++	+
114	MBF484	*Lactobacillus reuteri*	Infant feces	0.26	4.76	+++	+
115	MBF496	*Lactobacillus reuteri*	Infant feces	0.24	5.71	+++	++
116	MBF497	*Lactobacillus reuteri*	Infant feces	0.27	4.88	+++	+
117	MBF498	*Lactobacillus reuteri*	Infant feces	0.23	4.64	+++	+
118	MBF4112	*Lactobacillus reuteri*	Infant feces	0.67	7.28	+++	+
119	MBF4116	*Lactobacillus reuteri*	Infant feces	0.24	4.23	+++	+
120	NBF306	*Lactobacillus reuteri*	Infant feces	0.27	4.40	+++	+
121	NBF307	*Lactobacillus reuteri*	Infant feces	0.66	7.74	++	+
122	NBF308	*Lactobacillus reuteri*	Infant feces	0.76	8.32	++	+
123	NBF309	*Lactobacillus reuteri*	Infant feces	0.90	7.88	++	+
124	NBF310	*Lactobacillus reuteri*	Infant feces	0.57	7.17	++	+
125	NBF317	*Lactobacillus reuteri*	Infant feces	12.66	18.11	+	+
126	NBF319	*Lactobacillus reuteri*	Infant feces	5.73	12.01	+	+
127	NBF320	*Lactobacillus reuteri*	Infant feces	8.06	13.97	+	+
128	NBF331	*Lactobacillus reuteri*	Infant feces	0.66	7.71	+++	+
129	NBF342	*Lactobacillus reuteri*	Infant feces	1.80	8.32	++	+
130	NBF344	*Lactobacillus reuteri*	Infant feces	2.10	8.96	++	+
131	NBF347	*Lactobacillus reuteri*	Infant feces	0.27	7.13	++	+
132	NBF409	*Lactobacillus reuteri*	Infant feces	0.09	5.73	+++	+
133	NBF430	*Lactobacillus reuteri*	Infant feces	0.10	5.80	+++	+
134	NBF456	*Lactobacillus reuteri*	Infant feces	0.10	5.50	+++	+
135	NBF474	*Lactobacillus reuteri*	Infant feces	0.66	9.50	++	++

^a^1,3-PDO production:+++; > 12 g/L,++; > 7 g/L,+; > 0 g/L, –; not detected.

^b^Reuterin production:+++; > 0.5 at 560 nm,++; > 0.3 at 560 nm,+; > 0.1 at 560 nm, –; not detected.

In addition, the production of 1, 3-propanediol (1, 3-PDO) by the isolated *L. reuteri* strains was determined using a high-performance liquid chromatography (HPLC) system (Table 1 and Supplementary Fig. S1A). The 1, 3-PDO is valuable compound used as a replacement for petroleum-based glycols, including propylene glycol, butylene glycol, and glycerin. It has been reported that *L. reuteri* metabolizes glycerol to reuterin (3-hydroxypropionaldehyde, 3-HPA) and then converts reuterin to 1, 3-PDO. *L*. *reuteri* cannot grow on glycerol as the sole carbon source and the conversion to 1, 3-PDO from glycerol requires NADH produced by glucose metabolism. Therefore, we tested the production level of 1, 3-PDO under co-fermentation in the presence of both glycerol and glucose in the culture medium.

Interestingly, most (but not all) *L. reuteri* isolates originating from swine did not produce 1, 3-PDO, whereas every strain of human origin did (Table 1). Genomic analysis showed that *L. reuteri* AN417 isolated from swine did not encode glycerol dehydratase (*dhaB*), which catalyzes 3-HPA production, and 1,3-propanediol dehydrogenases (*dhaT*), which catalyze 1,3-PDO production. The data implied that the *L. reuteri* strains exhibit host-specific characteristics in metabolite production.

### Potentially important inhibitory activity of *L. reuteri* AN417 supernatant (LRS) against oral bacterial pathogens

When the antimicrobial activity of the newly identified *L. reuteri* strains was assessed, we observed that *L. reuteri* AN417 culture supernatant (LRS) outperformed those of our other tested strains. We first observed whether isolated *L. reuteri* strains affected the growth of the oral pathogens *S. mutans* KCTC 3065 and *P. gingivalis* BAA-308 (Fig. 1A, B). The clear zone was the largest when *S. mutans* was treated with LRS compared to the supernatants from other strains. Moreover, the growth of *P. gingivalis* was more highly inhibited when 10% (v/v) LRS was added to the medium than when supernatants derived from other *L. reuteri* strains were treated. Furthermore, growth of *P. gingivalis* was most inhibited when 10% (v/v) LRS was added to the medium (Fig. 1B). The findings indicated that cell-free culture supernatant derived from *L. reuteri* AN417 exhibited the highest activity against *P. gingivalis* and potentially against other oral pathogenic bacteria.

Next, we determined that the antimicrobial bioactive substances are present in the culture supernatant, not inside the bacterial cells themselves. To establish whether the active substances were present in the culture supernatant or in the bacterial cells, bacterial cell extracts (BE) were prepared using ethyl acetate. BE and LRS, which were concentrated and diluted to the desired values, were treated with *P. gingivalis* for 24 h. Compared with the control treated only with HEPES or de Man Rogosa and Sharpe (MRS), LRS substantially inhibited the growth of *P. gingivalis.* However, no effect was observed with the BE treatment. Furthermore, compared with control treatments, LRS significantly reduced *P. gingivalis* intracellular ATP levels, whereas the BE treatment did not (Fig. 1C). Administration of 20% or 40% (v/v) LRS for 96 h significantly reduced ATP levels and growth of the pathogenic bacteria (Fig. 1D).

### LRS inhibits the growth of oral pathogenic bacteria

**Figure 2:**
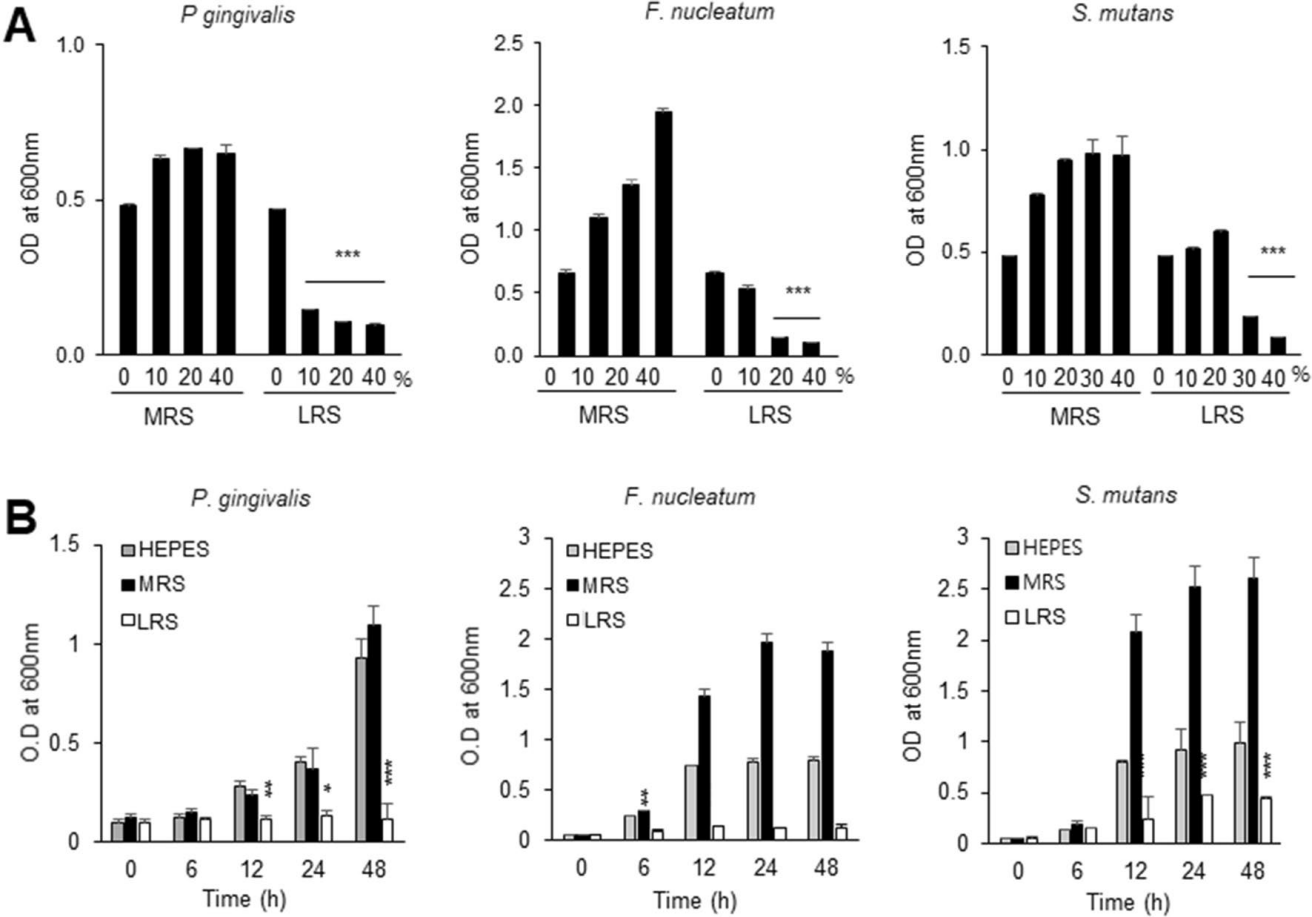
Effect of LRS on the growth of *P. gingivalis*, *F. nucleatum*, and *S. mutans*. (**A**) The effect of 0% (v/v), 10% (v/v), 20% (v/v), and 40% (v/v) LRS on the growth of pathogenic oral bacteria. Pure MRS medium was used as the control. Data presented are OD_600_ values after incubation for 24 h. Significant differences from the control are indicated by (**) for *p* < 0.01 and (***) for *p* < 0.001. (**B**) The effect of 40% (v/v) LRS on the growth of pathogenic oral bacteria over time. Pure MRS medium was used as the control. Data presented are culture OD_600_ values. Significant differences from the control are indicated by (*) for *p* < 0.05, (**) for *p* < 0.01, and (***) *p* < 0.001.

The inhibitory effects of 10%, 20%, 30%, and 40% (v/v) LRS on the growth of selected oral pathogenic bacteria (*P. gingivalis*, *F. nucleatum*, and *S. mutans*) were assessed. As the results, growth inhibition against tested pathogens was dependent on LRS concentrations. Treatment with 40% (v/v) LRS significantly inhibited the growth of the three pathogenic bacterial strains. In contrast, 10% (v/v) LRS did not inhibit the growth of *F. nucleatum* and *S. mutans*. However, the growth of *P. gingivalis* was significantly inhibited in 10, 20 and 40% of LRS treatment (80% reduction in growth rate) (Fig. 2A). Prolonged treatment for up to 48 h with 40% (v/v) LRS resulted in a stable continuation of the inhibitory effect in all three strains (Fig. 2B).

### LRS effectively reduces the viability of oral pathogenic bacteria

**Figure 3:**
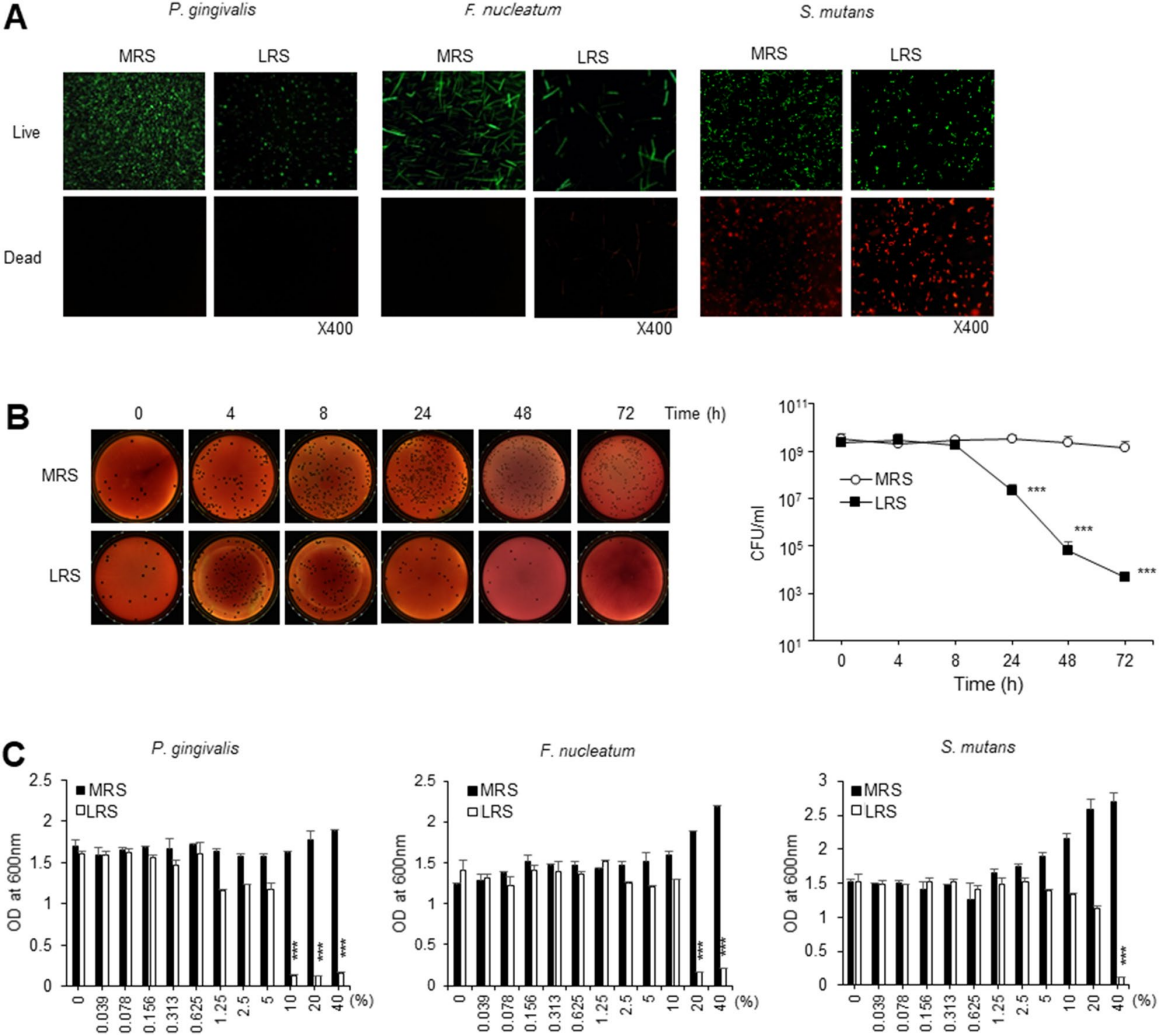
Analysis of the MIV of LRS and the effect of LRS on *P. gingivalis, F. nucleatum,* and *S. mutans* cell viability. (**A**) Representative fluorescence images of LIVE/DEAD BacLight viability assay of pathogenic bacteria exposed to 20% (v/v) LRS or MRS broth (control) for 24 h at 37 °C. Red fluorescence indicates dead or membrane-damaged bacterial cells and green fluorescence indicates live/healthy bacteria. Original magnification, ×400. (**B**) Quantification of *P. gingivalis* cell concentrations (CFU/mL) following treatment with 20% (v/v) MRS (control) or LRS. Black colonies developed after 6 days growth in a 37 °C anaerobic chamber. The time required to kill *P. gingivalis* treated with MRS or LRS is shown graphically. Significant differences from the control (*p* < 0.001) are indicated by ***. (**C**) MIV of LRS for pathogenic bacteria, determined based on OD_600_ values after 48 h incubation in an aerobic or aerobic chamber maintained at 37 °C. Significant differences from the control (*p* < 0.001) are indicated by ***.

In fluorescent cell-staining assays, LRS increased the number of dead pathogenic bacteria and decreased the number of live pathogenic bacteria. To evaluate the effect of LRS on the viability of the three pathogenic bacterial strains, a mixture of SYTO9 green fluorescence nucleic acid stain and propidium iodide was used for cell staining. The cells were observed by fluorescence microscopy. Compared with the negative control (MRS or Brain Heart Infusion [BHI] medium), the intensity of green fluorescence emitted by live bacteria decreased in *P. gingivalis, F. nucleatum,* and *S. mutans* cultures treated with LRS. In *S. mutans*, the intensity of red fluorescence, indicating dead bacteria, increased with LRS treatment (Fig. 3A). After treatment with LRS, bacterial death was observed over time. As a result, it was observed that *P. gingivalis* died rapidly starting 8 h after LRS treatment (Fig. 3B). These results were consistent with those of LIVE/DEAD BacLight analysis, with LRS treatment substantially reducing colony forming units compared with the MRS treatment.

### Antimicrobial activity of LRS against oral pathogenic bacteria, especially *P. gingivalis*

Treatment of the three oral pathogenic bacteria with LRS revealed a minimum inhibitory volume (MIV) of approximately 10% (v/v) for *P. gingivalis*. The MIV was 20% and 40% (v/v) for *F. nucleatum* and *S. mutans,* respectively (Fig. 3C).

### LRS impedes biofilm formation by the oral pathogenic bacteria

**Figure 4:**
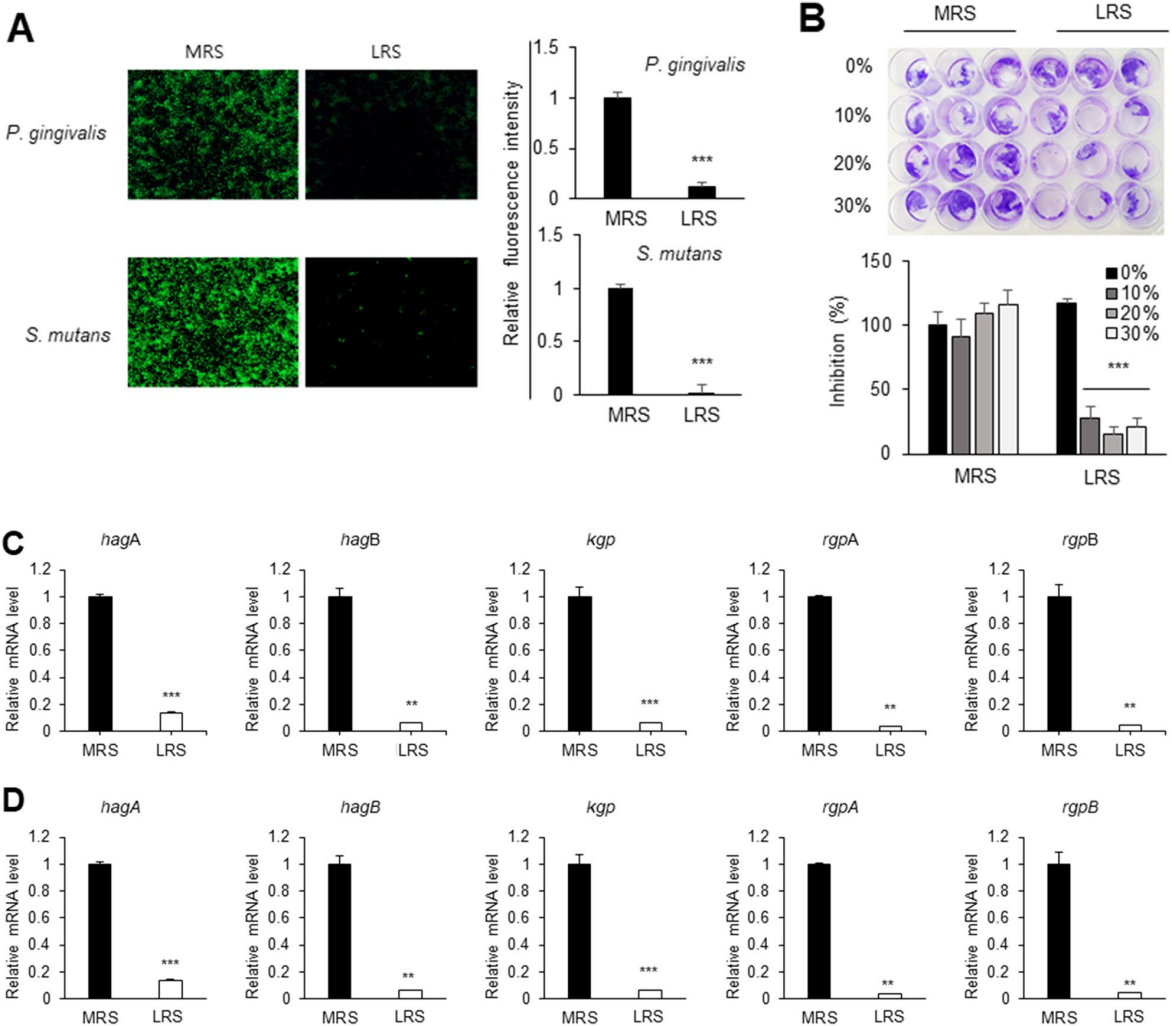
Activity of LRS against *P. gingivalis* and *S. mutans* biofilm formation (**A**) Visualization using fluorescence microscopy (left panel) of the antibiofilm effect of LRS. Non-adherent bacteria were removed, and the biofilms stained using the LIVE/DEAD BacLight Bacterial Viability Kit. Green and red fluorescence indicates live and dead bacteria, respectively. The fluorescence intensity ratio of live/dead cells (right panel) was analyzed using ImageJ software (IJ version 1.46r; https://imagej.nih.gov/ij/download.html). Significant differences from the control (*p* < 0.001) are indicated by ***. (**B**) Images showing crystal violet-stained biofilms of *P. gingivalis* following treatment with various concentrations of MRS (control) and LRS. Significant differences from the control (*p* < 0.001) are indicated by ***. (**C**,**D**) The effect of LRS on the expression of genes involved in biofilm formation. Bacteria were treated with MRS broth (control) or LRS for 48 h following (**C**) initial biofilm colonization or (**D**) following established biofilm formation (5 days). The mRNA levels of *hagA, hagB, rgpA, rgpB,* and *kgp* genes were quantitatively measured using RT-qPCR. The data are expressed as the relative level of 16S rRNA. Significant differences from the control are indicated by (**) *p* < 0.01 and (***) *p* < 0.001.

To confirm the antibiofilm activity of LRS against the biofilm formation during the early stage of bacterial colonization, LRS was added to *P. gingivalis* and *S. mutans* cultures immediately after bacterial inoculations, so that the effects of LRS on biofilm formation during the initial attachment phase could be examined. LRS treatment substantially reduced the fluorescence intensity compared with the control treatment (Fig. 4A), which was consistent with the quantitative results. To determine the concentration of LRS that eradicates established *P. gingivalis* biofilms, biofilms developed for 5 days were treated with LRS and stained with crystal violet. Compared with the control treatment, 10%, 20%, and 30% (v/v) treatment of LRS achieved substantial removal of biofilms (Fig. 4B). Furthermore, we observed a significant reduction in the expression of *rgpA*, *rgpB*, *hagA*, *hagB,* and *kgp*, all of which are genes involved in biofilm attachment and formation, in *P. gingivalis* following LRS treatment (Fig. 4C,D).

### Whole genomic sequencing of *L. reuteri* AN417

To determine the antibacterial and antibiofilm molecules produced by *L. reuteri* AN417, whole genome sequencing was performed using PacBio RSII single-molecule real-time (SMRT) sequencing technology. As shown in Supplementary Fig. S2, the complete genome consisted of a single circular chromosome of 2,069,421 bp and four circular plasmids of 93,397 bp (pLreu417A of 57,676 bp, pLreu417B of 16,368 bp, pLreu417C of 10,268 bp, and pLreu417D of 9085 bp) with 38.95% G + C content. A total of 2151 genes were predicted in the genome of this strain. Of these, 2034 were identified as protein-coding genes. The total length of the coding regions was 1,850,478 bp. A total of 1571 protein-coding genes were assigned putative functions. The remainder were annotated as hypothetical proteins.

### Genomic analysis and characterization of *L. reuteri* AN417

**Figure 5:**
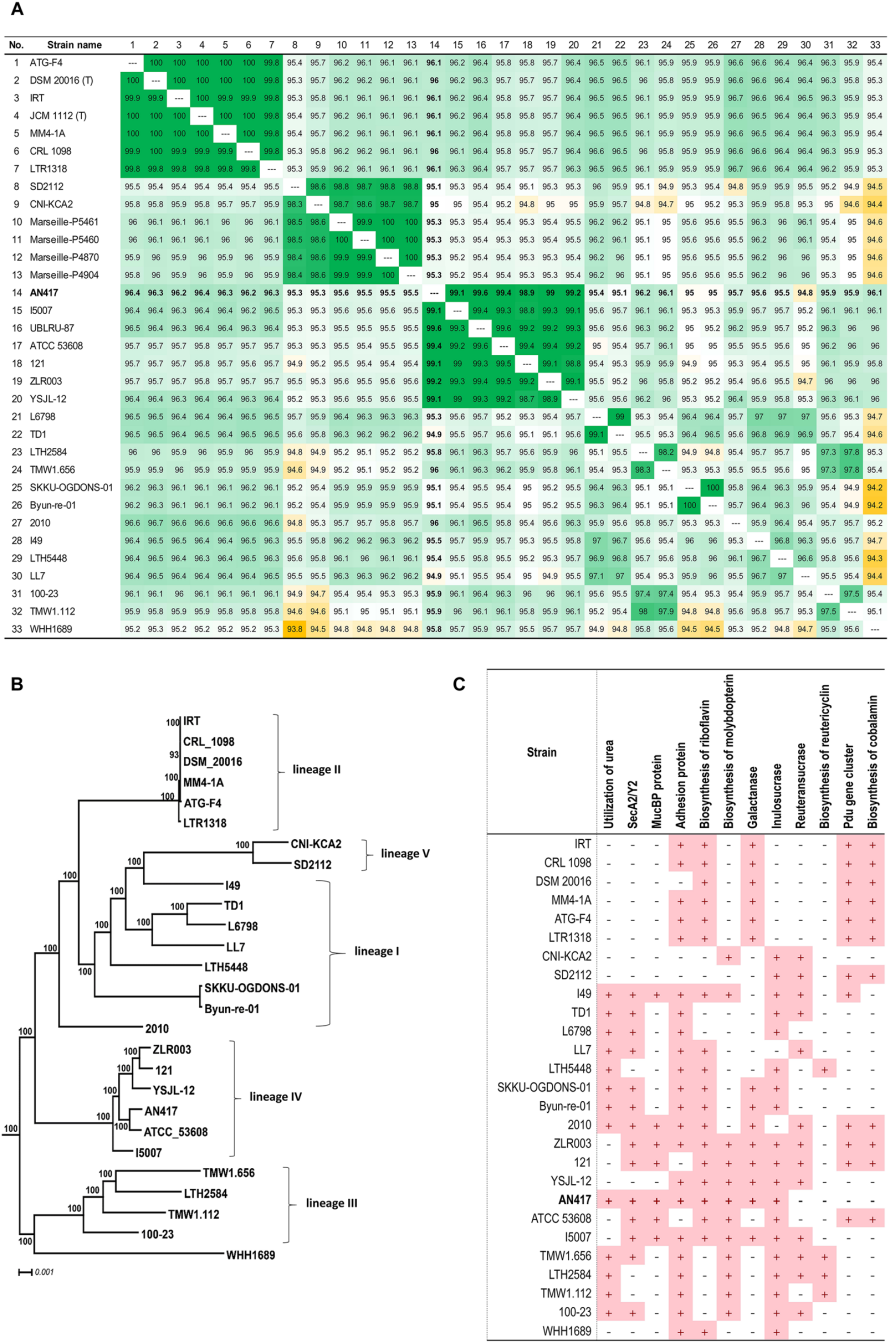
ANI values between genomes of *L. reuteri* strains and their phylogenetic position. (**A**) ANI values between genomes of *L. reuteri* strains. (**B**) Phylogenetic tree reconstructed using the amino acid alignments of 766 core genes using the maximum likelihood approach. Numbers above branches show maximum likelihood bootstrap supports from 500 non-parametric replicates. The tree was rooted using *L. gastricus* LG045 and *L. secaliphilus* DSM 17896 as outgroups. The scale bar represents the number of substitutions per site. (**C**) Genes encoding proteins related to environmental adaptation and antimicrobial compounds.

Genome-genome relatedness of *L. reuteri* AN417 was also analyzed by calculating the average nucleotide identity (ANI) and constructing a phylogenomic tree of 31 genome sequences with fewer than 30 scaffolds among the genomes of *L. reuteri* strains from the GenBank/EMBL/DDBJ database. *L. reuteri* genomes were an average size of 2.10 Mb and G + C ratio of 38.43–39.31 (Table 2). The whole genome of *L. reuteri* AN417 showed ANI values ranging from 94.8 to 99.6% with *L. reuteri* strains. The highest ANI values were obtained for the pig strains (Fig. 5A). Additionally, whole genome phylogenetic analysis was performed with 27 *L. reuteri* genomes, excluding those with many contigs. A phylogenetic tree of *L. reuteri* strains was constructed using the amino acid alignments of 766 core genes using the maximum likelihood approach. The tree showed clear separation of *L. reuteri* strains into five host-defined phylogenetic lineages (Fig. 5B). This analysis also revealed that *L. reuteri* AN417 isolated from pigs clustered in lineage IV, which is contained in strains originating from pigs.

**Table 2:** *Lactobacillus reuteri* genomes used for phylogeny reconstruction and comparative genomics.

Species	Strains	Genome assembly acc. No	Countries	Origin	No. of contigs	Genome size (bp)	GC ratio (%mol)	Notes
*L. reuteri*	121	GCA_001889975.1	Netherlands	Pig	14	2,302,234	39.01	–
*L. reuteri*	2010	GCA_003703885.1	USA	Rat	38	2,220,255	38.53	–
*L. reuteri*	100–23	GCA_000168255.1	New Zealand	Rat	2	2,305,557	38.73	–
*L. reuteri*	ATCC 53,608	GCA_000236455.2	Sweden	Pig	3	2,091,243	38.75	–
*L. reuteri*	ATG-F4	GCA_004208615.1	Korea	Human	1	2,041,516	38.89	–
*L. reuteri*	Byun-re-01	GCA_003316895.1	Korea	Mouse	1	2,244,514	38.88	–
*L. reuteri*	CNI-KCA2	GCA_012275185.1	Nigeria	Chicken	1	2,072,001	38.92	–
*L. reuteri*	CRL 1098	GCA_001657495.1	Germany	Sourdough	45	1,963,029	38.74	–
*L. reuteri*	DSM 20,016	GCA_000016825.1	–	Human	1	1,999,618	38.87	Type strain
*L. reuteri*	I49	GCA_001688685.2	Switzerland	Mouse	1	2,044,771	38.76	–
*L. reuteri*	I5007	GCA_000410995.1	China	Pig	7	2,093,275	38.93	–
*L. reuteri*	IRT	GCA_001046835.1	Korea	Human	1	1,993,967	38.90	–
*L. reuteri*	L6798	GCA_900093565.1	Sweden	Mouse	35	2,108,374	38.43	–
*L. reuteri*	LL7	GCA_007633215.1	USA	Mouse	2	2,384,717	38.81	–
*L. reuteri*	LTH2584	GCA_000712555.1	Germany	Sourdough	55	2,066,054	38.53	–
*L. reuteri*	LTH5448	GCA_000758185.1	Germany	Sourdough	36	1,980,298	38.44	–
*L. reuteri*	LTR1318	GCA_009184725.1	China	Human	2	2,047,619	38.98	–
*L. reuteri*	Marseille-P4870	GCA_901600665.1	France	Yogurt	287	2,039,591	38.98	For only ANI calculation
*L. reuteri*	Marseille-P4904	GCA_901600705.1	France	Yogurt	282	2,039,591	38.98	For only ANI calculation
*L. reuteri*	Marseille-P5460	GCA_901600695.1	France	Yogurt	316	2,039,540	39.21	For only ANI calculation
*L. reuteri*	Marseille-P5461	GCA_901600675.1	France	Yogurt	311	2,039,572	39.21	For only ANI calculation
*L. reuteri*	MM4-1A	GCA_000159475.2	Finland	Human	7	2,067,914	38.88	–
*L. reuteri*	SD2112	GCA_000159455.2	Peru	Human	5	2,316,838	39.04	–
*L. reuteri*	SKKU-OGDONS-01	GCA_003316935.1	Korea	Chicken	1	2,259,968	38.86	–
*L. reuteri*	TD1	GCA_000439275.1	USA	Rat	1	2,145,445	38.78	–
*L. reuteri*	TMW1.112	GCA_000722535.2	Germany	Sourdough	12	2,032,034	38.45	–
*L. reuteri*	TMW1.656	GCA_000712565.2	Germany	Sourdough	17	1,949,539	38.49	–
*L. reuteri*	UBLRU-87	GCA_003719715.1	India	Fermented food	91	1,821,307	38.70	For only ANI calculation
*L. reuteri*	WHH1689	GCA_003072625.1	China	Highland barley wine	1	2,044,184	39.31	–
*L. reuteri*	YSJL-12	GCA_006874665.1	China	Pig	3	2,151,788	38.93	–
*L. reuteri*	ZLR003	GCA_001618905.1	China	Pig	1	2,234,097	38.66	–
*L. reuteri*	AN417	In this study	Korea	Pig	5	2,162,818	38.95	–
*L. gastricus*	LG045	GCA_009648555.1	Korea	Human	3	1,905,155	41.65	Outgroup for tree
*L. secaliphilus*	DSM 17,896	GCA_001437055.1	Germany	Sourdough	15	1,646,143	47.72	Outgroup for tree

Whole genome phylogenetic analysis was performed with 27 *L. reuteri* genomes except for them with large numbers of contigs.

The whole genome sequence of *L. reuteri* AN417 encodes the urease complex UreABCEFGD unlike general strains isolated from human and pigs, contributing to its viability under acidic conditions (Fig. 5C). The mucin binding protein, Muc2, contributes to host adaptation and adhesion to mucus. However, genes encoding reuteran, reutericyclin*,* and reuterin, which are important in the antimicrobial activity of *L. reuteri* strains, were absent in the genome (Fig. 5C). In addition, reuterin production was also measured in *L. reuteri* AN417 (Table 2), demonstrating that *L. reuteri* AN417 did not produce reuterin. This result was consistent with the genomic analysis results (Table 1).

### Antibacterial activity of LRS is mediated by the presence of fatty acids and sugars

**Figure 6:**
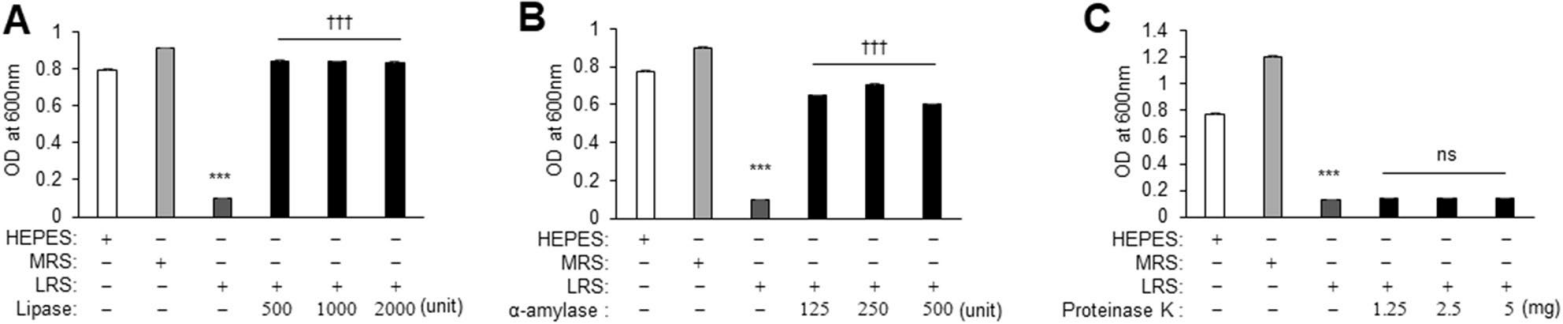
Exploring antibacterial substances in LRS. (**A**–**C**) Antibacterial effects of LRS and enzyme-treated (α-amylase, lipase, and proteinase) LRS against *P. gingivalis* after a 24 h incubation. Significant differences from control and LRS treatments (*p* < 0.001) are indicated by (***) and (^†††^), respectively.

To categorize the type of metabolites responsible for the activity of LRS against periodontopathogens, the antibacterial effect of LRS against *P. gingivalis* in the presence of proteinase K, lipase, and α-amylase was evaluated. Treatment of LRS with lipase or α-amylase eliminated the inhibitory effect of LRS on *P. gingivalis* growth (Fig. 6A–C). These findings suggested that the antibacterial effect of LRS against oral pathogenic bacteria could be attributed to the presence of a fatty acid and/or sugar.

## Discussion

The present study reports the antibacterial effects of cell-free culture supernatant from *L. reuteri* strain AN417 (LRS), a strain that was isolated from the porcine small intestine, against selected oral pathogenic bacteria. Based on our results, we anticipate that *L. reuteri* AN417 can positively affect oral health. Recently, there has been growing interest in the potential and utilization of microbial metabolites, termed postbiotics^23^. The cell-free culture supernatant of *L. reuteri* AN417 exhibited greater antimicrobial activity than those of the known *Lactobacillus* reference strains KCTC 3594 and KCTC 3678, which also inhibited periodontopathic bacteria (Fig. 1A,B). In our study, multiple lines of scientific evidence validated the antimicrobial activity of LRS.

Evaluation of the antimicrobial effects of naturally-derived agents has largely focused on their activity against *P. gingivalis, F. nucleatum,* and *S. mutans* because these bacteria have received the most attention in relation to oral diseases and are implicated in periodontal diseases, dental caries, and endocarditis^7–10^. To date, the antimicrobial effects of naturally derived agents against these bacteria have been evaluated in numerous studies, with a recent study reporting the antimicrobial activities of *L. reuteri* supernatant against *P. gingivalis*^21^. Hence, we also focused on these three oral pathogens in our study.

The major antibacterial compounds produced by *L. reuteri* strains are reuterin, reuteran, and reutericyclin. Genomic analysis of *L. reuteri* AN417 revealed the absence of a *pdu*-*cbi*-*cob*-*hem* gene cluster for the biosynthesis of reuterin and cobalamin (vitamin B_12_), and genes for the synthesis of reutericycin and reuteran. However, *L. reuteri* AN417 encoded an inulin-type fructansucrase. The strain also encoded the UreABCEFGD urease complex, which was not found in the other strains isolated from humans and pigs, contributing to its viability under acidic conditions.

We compared the activity of concentrated *L. reuteri* AN417 cell-free culture medium (LRS) to the activity of *L. reuteri* AN417 cell extracts using organic solvents. The results showed that *L. reuteri* AN417 cell extracts had no antimicrobial activity, whereas LRS did (Fig. 1C). From these data, secondary metabolites produced during growth are thought to play a crucial role in antimicrobial activities against oral pathogenic bacteria^24^. Bungenstock et al. reported that the antibacterial effect of probiotics against fermented foodborne pathogens is attributable to their production of lactic acid and the associated increase in acidity^25^. However, the antimicrobial activity of LRS observed in this study may not have been due to lactic acid alone. Probiotic *Lactobacillus* spp. are also known to produce various metabolites that defend against *S. mutans* colonization^26^. The level of intracellular ATP, the energy source for viable oral pathogenic bacteria, was reduced significantly by LRS, leading to the growth inhibition of oral pathogenic bacteria (Fig. 1D, right). The inhibitory effect was maintained for 96 h, indicating that the bioactive substances in the LRS were stable over time. Growth of *S. mutans* (a Gram-positive bacterium)*, F. nucleatum* (Gram-negative)*,* and *P. gingivalis* (Gram-negative) was inhibited by adding 30% (v/v), 20% (v/v), and 10% (v/v) of LRS, respectively (Fig. 2A), demonstrating that bacterial growth inhibition by LPS was the most potent and most specific for *P. gingivalis.* In addition, we speculate that higher concentrations of antimicrobial substances are required to inhibit the growth of Gram-positive bacteria, which have thick peptidoglycan cell walls.

Endotoxins, such as lipopolysaccharides (LPS), are produced from oral bacterial biofilms, including oral plaques. Endotoxins destroy alveolar bone and induce a series of inflammatory reactions that ultimately lead to tooth loss^27^. To determine whether the effect originated from bacterial membrane damage, SYTO9 and propidium iodide staining was performed. LRS treatment resulted in a marked decrease in viable bacterial cells (Fig. 3). This result may imply that bacterial membrane integrity was weakened by treatment with LRS, which led to the inhibition of bacterial growth and a reduction in the continuous release of endotoxins.

Biofilms are the cause of dental bacterial infections^28,29^. Biofilms are comprised of extracellular polymeric substances secreted by bacteria during metabolic processes. The biofilm structure confers antibiotic resistance^30^. Biofilms in the oral cavity generally contribute to periodontitis. Within biofilms, resident microbes are resistant to external attacks that include antibacterial agents, and the bacteria dispersed from the biofilms can cause infection^31^. Biofilm dispersal agents might be the most suitable targets for the prevention of periodontitis and dental caries. In previous studies, supernatants from cultures of *Lactobacillus* sp. inhibited biofilm formation and reduced the expression of genes related to the production of exopolysaccharides^26^. In the current study, LRS significantly reduced biofilm formation (Fig. 4), which could contribute to the prevention of dental caries and periodontitis.

LRS has excellent inhibitory abilities against the growth and biofilm formation of the tested bacteria. In particular, *P. gingivalis* required the lowest amounts of LRS to inhibit its growth and biofilm formation among the bacteria tested in our study. Thus, we focused on *P. gingivalis* in our other analyses, as this was the pathogen most strongly affected by LRS.

The antimicrobial activity of *L. reuteri* has been attributed to its production of organic acids, hydrogen peroxide, and bacteriocin-like compounds^32^, such as reuterin, reuteran, and reutericyclin^33^. However, *L. reuteri* AN417 lacks the ability to produce them because of the absence of the required genes. Thus, to identify the antimicrobial substance in LRS, various enzymes such as α-amylase, lipase, and proteinase were added to LRS to catabolize and inactivate any sugars, lipids, or proteins that could confer antimicrobial activity. To confirm which enzymes cause loss of activity, each LRS treated with enzymes was added to *P. gingivalis* cultures. LRS treated with lipase and α-amylase did not inhibit *P. gingivalis* growth, suggesting that the active substance responsible for LRS antibacterial activity was either a fatty acid or a sugar. Although *L. reuteri* reportedly produces antimicrobial molecules, including lactic acid, acetic acid, ethanol, and reutericyclin^34^, fatty acid and sugar-based antimicrobial substances have not yet been reported.

According to a previous study^35^, soluble or immobilized PLNC8 αβ bacteriocins from *L. plantarum* strains NC8 and 44048 prevent *P. gingivalis* colonization and pathogenicity. Therefore, it is necessary to conduct additional experiments under various conditions to support the findings of this study. Furthermore, the bioactive substances in LRS should be identified and purified for practical use.

In this study, we show that supernatants derived from *L. reuteri* AN417 cultures are able to suppress the growth and biofilm formation of oral pathogenic bacteria. Interestingly, *L. reuteri* AN417 does not produce reuteran, reutericyclin*,* and reuterin, which are important in the antimicrobial activity of reported *L. reuteri* strains. Although further studies are required, the antibacterial substance in LRS is suspected to be a fatty acid or a sugar, which has not yet been reported. Thus, LRS has potential as a novel bioactive substance for the prevention and treatment of pathogens associated with periodontitis.

## Methods

### Strain isolation and identification

Novel *Lactobacillus reuteri* strains were isolated from seven infants aged 3–7 years and from the small intestines of 13 6-month-old female pigs in the Republic of Korea. The pigs were fed a mixed diet. The intestinal contents from each child or pig were resuspended and serially diluted in sterile 0.85% NaCl. Aliquots were cultured anaerobically on MRS agar in an atmosphere of CO_2_:H_2_:N_2_ (7:7:86) atmosphere. After 2–3 days, single colonies were subcultured on fresh MRS agar. All colonies were selected irrespective of their shape and size. Genomic DNA (gDNA) was extracted and purified from cells grown on MRS agar as described previously^36^. The gDNA was used for 16S rRNA gene amplification and sequencing, and whole genome sequencing. The complete 16S rRNA gene sequence was amplified using universal primers: 27F (5′-AGAGTTTGATCMTGGCTCAG-3′) and 1492R (5′-TACGGYTACCTTGTTACGACTT-3′). The amplified genes were sequenced and compared with sequences obtained from the EzBioCloud^37^ and GenBank/EMBL/DDBJ (http://www.ncbi.nlm.nih.gov/blast) databases.

### Identification of isolates using a MALDI-TOF Biotyper

One colony of each bacterial isolate was subcultured for 24 h and used for MALDI-TOF Biotyper analysis. The colony was acquired using a toothpick and spotted onto a polished steel MALDI target plate. One microliter of formic acid (70% in water) was added to the spot and dried. Subsequently, 1 µL of MALDI matrix (10 mg/mL solution of α-cyano-4-hydroxycinnamic acid (HCCA) in 50% acetonitrile/2.5% trifluoroacetic acid) was added to the spot and dried. The MALDI target plate was placed in the MALDI-TOF/Microflex LT instrument (Bruker Daltonics, Billerica, MA, USA) for automated measurement and data interpretation. The MALDI Biotyper output is a log (score) between 0 and 3.0, which is calculated from a comparison of the peak list from an unknown isolate with the reference MSP in the database. A log (score) ≥ 1.7 was indicative of a close relationship at the genus level. A log (score) ≥ 2.0 was set as the threshold for a match at the species level. Isolates with a log (score) ≥ 2.0 were accepted as the correct identification.

### Bacteria, media, and culture

*Lactobacillus reuteri* strains KCTC 3594, KCTC 3678, KCTC 3679, KCTC 3680, KCTC 3682, and KCTC 3683 were used as reference strains. All samples were purchased from the Korean Collection for Type Cultures (KCTC, Daejeon, Korea). Reference strains and newly isolated *L. reuteri* strains (AN417, AN306, AN403, AN413, AN507, AN509, AN510, AN511, AN513, AN519, AN523, AN705, AN711, and RI-7) were grown on MRS agar plates. *P. gingivalis* strain BAA-308 and *F. nucleatum* KCTC 15573 were purchased from the American Type Culture Collection (Manassas, VA, USA) and KCTC, respectively. They were grown in trypticase soy broth (TSB; BD, Germany) comprised of (per L): 30 g trypticase soy broth, 5 mg hemin, 5 g yeast extract, 1 mg vitamin K1, and 15 g agar, or blood TSB agar (TSB medium plus 15 g/L agar and supplemented with 3% sheep blood). For all experiments, *P. gingivalis* and *F. nucleatum* were also cultured in TSB broth for at least 12 h prior to inoculation. Bacteria were grown and maintained at 37 °C in an anaerobic chamber in an atmosphere of CO_2_:H_2_:N_2_ (5:10:85). *S. mutans* (KCTC 3065) strains were grown in brain heart infusion (BHI) medium at 37 °C under aerobic conditions.

### Preparation of the test sample

*Lactobacillus reuteri* AN417 was grown in MRS broth for 48 h at 37 °C under anaerobic conditions to produce stationary phase cultures. The supernatant from *L. reuteri* AN417 culture (LRS) was collected via centrifugation at 4 °C for 30 min at 1500×*g*. The LRS was filtered through a 0.2 μm membrane filter to remove the remaining bacteria and debris. *L. reuteri* AN417 cell extract (BE) was prepared by the addition of 0.3 L ethyl acetate to the cell pellet, stirring the mixture for 24 h, and passing the mixture through a Phenex Teflon Polytetrafluorethylene filter membrane. After centrifugation at 1500×*g* for 20 min, the cell pellet was removed. The cell-free supernatant and BEs were concentrated to 20 × using a rotary evaporator and stored at 4 °C until required.

### Determination of 1, 3-PDO production

*Lactobacillus reuteri* strains were grown in MRS medium (10 g/L proteose peptone No. 3, 10 g/L beef extract, 5 g/L yeast extract, 20 g/L dextrose, 1 g/L polysorbate 80, 2 g/L ammonium citrate, 5 g/L sodium acetate, 0.2 g/L MgSO_4_, 0.05 g/L MnSO_4_, and 2 g/L dipotassium sulfate) supplemented with 20 g/L glycerol for 12 h under anaerobic conditions. The concentration of 1,3-PDO was determined using a 1200 series HPLC system (Agilent, Santa Clara, CA, USA) with a refractive index detector (RID) and an ion-exchange column (Aminex HPX-87H; Bio-Rad, Hercules, CA, USA). The mobile phase was 2.5 mM H_2_SO_4_, flow rate was 0.6 mL/min, column temperature was 65 °C, and RID was maintained at 45 °C.

### Measurement of the concentration of reuterin (3-HPA)

Cultivation to stationary phase was performed in a 250 mL round-bottom flask containing 100 mL of MRS medium for 24 h at 37 °C. Cells were harvested by centrifugation at 16,000×*g* for 5 min and washed twice in 50 mM sodium phosphate buffer (pH 7.5). The washed cells were resuspended in 200 mM glycerol solution and incubated at 30 °C for 3-HPA production. To measure the 3-HPA conversion yield, samples were collected at 1 h intervals. The samples were centrifuged at 16,000×*g* for 5 min and the supernatant was used to determine 3-HPA content. A previously described colorimetric method^38^ was used to determine the content of 3-HPA in the samples. The reaction consisted of 1 mL of the sample, 3 mL of HCl (37%), and 0.75 mL of 10 mM tryptophan-HCl. The mixture was mixed and incubated at 37 °C for 20 min, and the absorbance at 560 nm was measured using a SpectraMax 190 ELISA reader (Molecular Devices Corp., Sunnyvale, CA, USA). The amount of 3-HPA was calculated from the absorbance at 560 nm with acrolein as a standard.

### Disk diffusion assay

Antimicrobial activity was determined using the disk diffusion assay. Cell-free supernatants of *L. reuteri* strains were prepared using 48-h cultures. Agar plates were inoculated with *E. coli, P. aeruginosa,* or *S. mutans.* Filter paper discs, approximately 6 mm in diameter, soaked with 50 µL of supernatant, were placed on the agar surface. After 24–48 h of incubation, the absence or presence of a clear zone around the disk was observed.

### Growth rate measurement

To study the growth rates of the selected pathogenic bacteria in the presence or absence of LRS, bacteria were grown in 1 mL of the appropriate medium containing different concentrations of LRS at 37 °C in an anaerobic chamber or orbital shaker. Growth rates were determined by measuring the culture optical density at 600 nm (OD_600_) at various times.

### ATP bioluminescence assay

ATP levels in cultures of the selected pathogenic bacteria treated with various concentrations of LRS were measured using a BacTiter-Glo ATP Assay Kit (Promega, Madison, WI, USA) according to the manufacturer’s instructions. Bioluminescence measurements were obtained in triplicate for each sample. Pure culture media were used as negative controls. Luminescence was measured using a luminometer (Promega).

### Minimal inhibitory volume (MIV) assay

The MIV of LRS was determined using a modified method of a previously described procedure^39^. Pathogenic bacterial cultures generated in suitable liquid culture media as detailed above were diluted with nutrient broth to an OD_600_ of 0.005 or 0.05. The diluted bacterial suspensions were then treated with either MRS medium (control) or 1 × LRS, dispensed into the first well of a 96-well plate, and serially diluted into consecutive wells. Plates were incubated at 37 °C for 24 h, and the absorbance at 600 nm was measured using a microplate reader.

### LIVE/DEAD *Bac*Light viability assay

The LIVE/DEAD BacLight Kit (Molecular Probes-Invitrogen, Carlsbad, CA, USA) was used to distinguish live and dead bacterial cells. The assay, which is based on membrane integrity and nucleic acid staining, was performed according to the manufacturer’s instructions. SYTO9 green fluorochrome (Thermo Fischer Scientific, Waltham, MA, USA) can penetrate the intact bacterial membrane, whereas the larger red fluorochrome, propidium iodide, only penetrates the membranes of damaged bacteria. Cells treated with LRS or MRS medium (control) for 24 h were stained in the dark for 15 min. The cells were mounted on slides and evaluated by fluorescence microscopy.

### Biofilm inhibition assay

The bacterial suspension was prepared by diluting an overnight culture of bacteria in TSB broth. Dilutions of LRS in bacterial suspensions were prepared in polystyrene-coated flat-bottomed 24-well plates and incubated at 37 °C for 5 days without shaking to allow the development of multilayer biofilms. A pure culture medium served as a negative control. Biofilm biomass was assayed using the modified crystal violet staining assay and a LIVE/DEAD BacLight Kit (Molecular Probes-Invitrogen, Carlsbad, CA, USA) assay.

### Time-kill assay

The time-to-kill *P. gingivalis* following LRS treatment was determined based on a previously described protocol^40^. A bacterial suspension (OD_600_ = 1) was treated with LRS and incubated anaerobically at 37 °C. An aliquot (100 μL) of this bacterial culture was removed at 0, 4, 8, 24, 48, and 72 h following LRS treatment, and plated on TSB agar to quantify the number of colony forming units (CFUs) in the treated cultures. Pure MRS medium was used as a negative control.

### Real-time quantitative polymerase chain reaction (RT-qPCR) analysis

The potential of LRS to prevent biofilm formation and/or destroy established biofilms was investigated. *P. gingivalis* (OD_600_ = 0.6) was dispensed into a polystyrene-coated 6-well plate and treated with MRS medium (control) or LRS for 24 h in an anaerobic incubator. In addition, *P. gingivalis* biofilms were established in a polystyrene-coated 6-well plate for 5 days, after which these biofilms were treated with either MRS medium (control) or LRS for 48 h in an anaerobic incubator. Total RNA was extracted with TRIzol reagent, and cDNA was synthesized using a PrimeScript RT reagent Kit (TaKaRa Bio, Shiga, Japan). RT-qPCR analysis of cDNA was performed according to the manufacturer’s instructions (Bioneer, Daejeon, South Korea) to investigate the mRNA expression of the selected genes related to biofilm formation. The following primers were used: *hag*A, Forward: 5′-ACAGCATCAGCCGATATTCC-3′, Reverse: 5′-CGAATTCATTGCCACCTTCT-3′; *hag*B, Forward: 5′-TGTCGCACGGCAAATATCGCTAAAC-3′, Reverse: 5′-CTGGCTGTCCTCGTCGAAAGCATAC-3′; *rgp*A, Forward: 5′-GCCGAGATTGTT CTTGAAGC-3′, Reverse: 5′-AGGAGCAGCAATTGCAAAG-3′; *rgp*B, Forward: 5′-CGCTGATGAAACGAACTTGA-3′, Reverse: 5′-CTTCGAATACCATGCGGT-3′; *kgp*, Forward: 5′-AGCTGACAAAGGTGGAGACCAAAGG-3′, Reverse: 5′-TGTGGCATG AGTTTTTCGGAACCGT-3′; and *16S* rRNA, Forward: 5′-TGTAGATGACTGATGGTG AAA-3′, Reverse: 5′-ACTGTTAGCAACTACCGATGT-3′. The reaction procedure involved incubation at 95 °C for 5 min followed by 30 cycles of 95 °C for 10 s, 55 °C for 30 s, and 72 °C for 30 s. Three independent reactions were conducted in triplicate for each gene.

### Effect of enzymes on antibacterial activity

*Lactobacillus reuteri* AN417 cell-free supernatants were treated with enzymes to evaluate the effect of enzymes on antibacterial substances. The LRS were treated with proteinase K (1 mg/mL), lipase (700 units/mg), or α-amylase (150 units/mg). For lipase and α-amylase treatment, the pH of the LRS was adjusted to 6.5 with NaOH to facilitate enzymatic activity. Enzymes were activated by incubation of the enzyme-treated supernatant at 37 °C for 3 h, after which the enzymes were immediately inactivated at 95 °C for 3 min. LRS-enzyme suspensions were centrifuged, and the supernatant was stored at 4 °C until further use.

### Whole genome sequencing and analysis

Whole genome sequencing of strain 417 was performed using PacBio RS II (Pacific Biosciences, Menlo Park, CA, USA) SMRT sequencing technology. A standard PacBio library with an average of 20 kb inserts was prepared and sequenced, yielding > 371.74 × average genome coverage. De novo assembly of the 128,664 subreads with 7,806 nucleotides on average (1,004,367,686 bp in total) was conducted using the hierarchical genome-assembly process pipeline in SMRT Analysis v2.3.0^41^. To correct the sequencing errors that can occur at both ends of a contig, the SMRT resequencing protocol was performed with assembly in which the first half of the contig was switched with the second half. Protein-coding genes were predicted using Prodigal v.2.6.3. Ribosomal RNA, transfer RNA, and miscellaneous features were predicted using Rfam v12.0^42^. CRISPR loci were predicted using the CRISPR recognition tool. ANI values were calculated using an online ANI calculator^43^.

### Statistical analyses

All statistical analyses were performed using Student’s t-test. The results are expressed as the mean ± standard deviation for each group. Multiple group data were analyzed using one-way analysis of variance, followed by Dunnett’s multiple range test. The threshold for significance was set at *p* < 0.05. Data shown are representative of three independent experiments, except for Fig. 1B, in which the data are from two independent experiments.

## Supplementary Information

Supplementary Figure Legends.

Supplementary Figure 1.

Supplementary Figure 2.

## Data Availability

Whole genome sequences were deposited with Bioproject PRJNA637956 and Biosample SAMN15162791, respectively. GenBank accession numbers are CP054657 for single chromosome and CP054658–CP054661 for the four plasmids, respectively.
