# Supplementary material for: Lactobacillus reuteri AN417 cell-free culture supernatant as a novel antibacterial agent targeting oral pathogenic bacteria
Source: Sci Rep. 2021 Jan 15;11:1631. doi: 10.1038/s41598-020-80921-x (PMC7810884; doi:10.1038/s41598-020-80921-x)
Supplement: Supplementary file 1 — Supplementary Figure Legends. [file 41598_2020_80921_MOESM1_ESM.docx]

***Lactobacillus reuteri* AN417 cell-free culture supernatant as a novel antibacterial agent targeting oral pathogenic bacteria**

Kyung Mi Yang^1, †^, Ji-Sun Kim^2, †^, Hye-Sung Kim^1^, Young-Youn Kim^1^, Jeong-Kyu Oh^1^, Hye-Won Jung^1^, Doo-Sang Park^2, *^, Kwang-Hak Bae^1, *^

**Figure Legend for Supplemental Figures**

**Supplemental Figure S1. Isolation of *L. reuteri* AN417 and 1,3-PDO production. (A)** Procedure of *L. reuteri* AN417 isolation and identification. (B) Determination of 1,3-PDO production by using HPLC. While *L. reuteri* SBF0331 produced 1,3-PDO, *L. reuteri* AN417 did not during fermentation.

**Supplemental Figure S2. Genomic features of *L. reuteri* AN417.** (A) Complete genome map and general genomic features of *L. reuteri* AN417. (B, C) Properties of one chromosome and four plasmids.
