# Supplementary figures and images for: Lactobacillus reuteri AN417 cell-free culture supernatant as a novel antibacterial agent targeting oral pathogenic bacteria
Source: Sci Rep. 2021 Jan 15;11:1631. doi: 10.1038/s41598-020-80921-x (PMC7810884; doi:10.1038/s41598-020-80921-x)

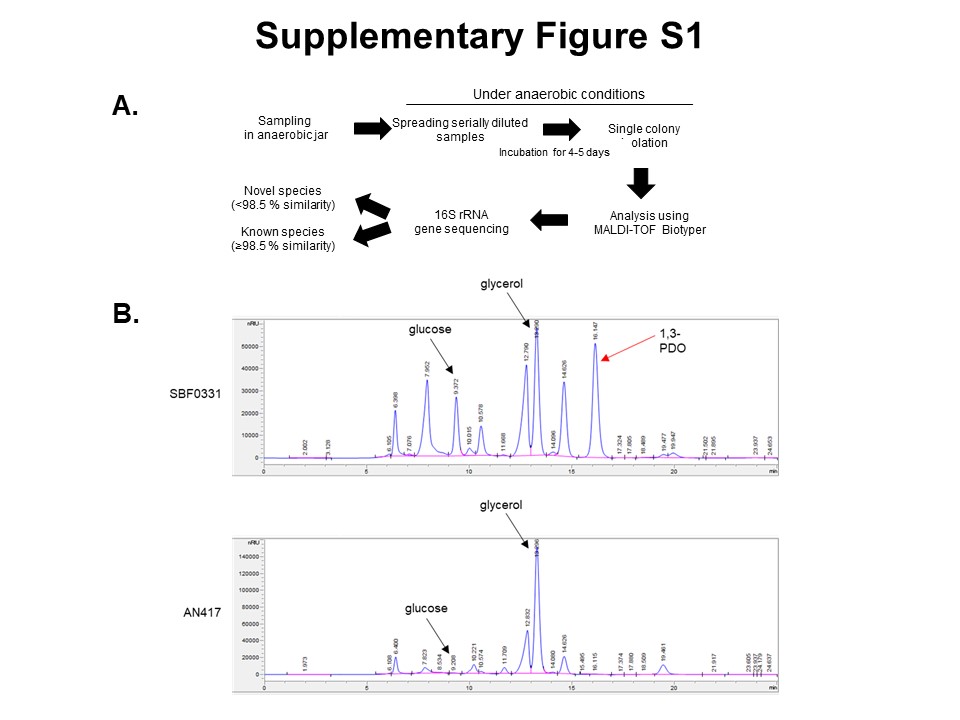

Supplement: Supplementary file 2 — Supplementary Figure 1. [file 41598_2020_80921_MOESM2_ESM.jpg]

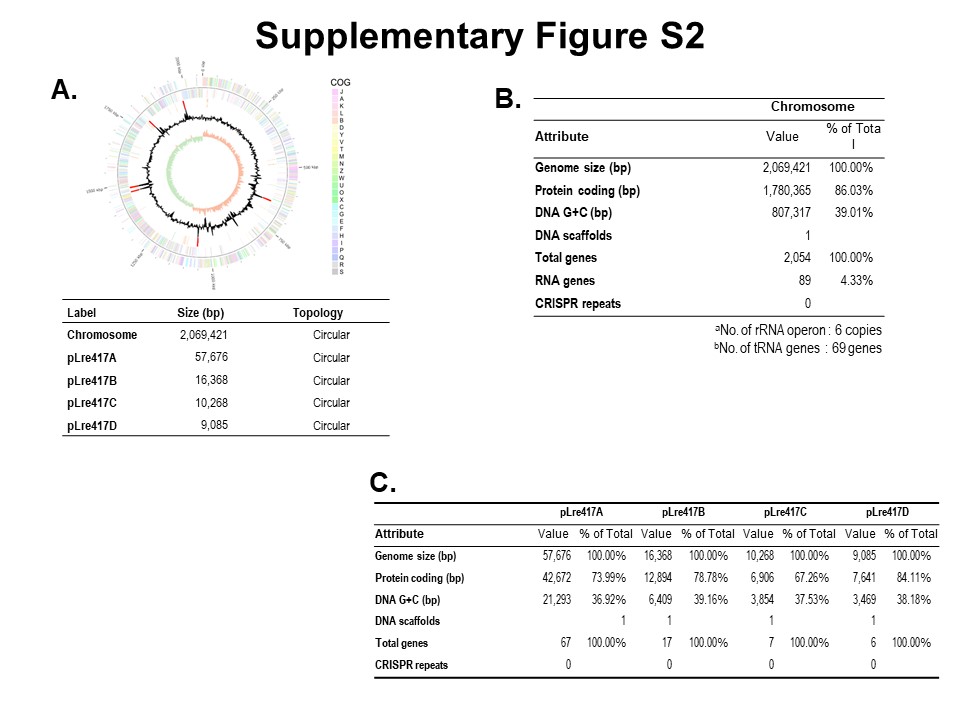

Supplement: Supplementary file 3 — Supplementary Figure 2. [file 41598_2020_80921_MOESM3_ESM.jpg]
